# Integrative transcriptome and metabolome evaluation of melanin biosynthesis in *Phyllostachys nigra* during low-temperature growth

**DOI:** 10.48130/forres-0025-0020

**Published:** 2025-09-23

**Authors:** Haojie Wang, Yadan Cao, Shawn D. Mansfield, Pengwei Zhang, Xinchun Lin, Dan Hou

**Affiliations:** 1 National Key Laboratory for Development and Utilization of Forest Food Resources, Zhejiang A&F University, Hangzhou 311300, China; 2 Key Laboratory of Bamboo Science and Technology of Ministry of Education, Bamboo Industry Institute, Zhejiang A&F University, Hangzhou 311300, China; 3 Department of Wood Science, University of British Columbia, Vancouver, BC V6T 1Z1, Canada; 4 Department of Botany, University of British Columbia, Vancouver, BC V6T 1Z1, Canada

**Keywords:** *Phyllostachys nigra*, Melanin, Low temperature, Transcriptome, Metabolome

## Abstract

Plant melanin is an organic molecule commonly used in medicine, food, and chemical industries. However, the molecular underpinnings of plant melanin's biosynthesis and its regulation are still unclear. *Phyllostachys nigra* is well known for its ornamental value because of its black culms. The black pigments enriched in the epidermis and cortex of *P. nigra* were identified to be melanin by analyses of its physical and chemical properties. Moreover, the biosynthesis of melanin was examined using comprehensive transcriptomic and metabolomic analyses in *P. nigra* when grown at low temperatures. Nontargeted metabolite profiling revealed that some indoles, including serotonin, 3-indoleacetic acid, and 1H-indole-3-acetamide, were significantly enriched in *P. nigra* when grown at low temperatures. Parallel transcriptomic analysis showed that a set of structural genes involved in serotonin biosynthesis was significantly upregulated by low temperatures. By integrating the transcriptome data and weighted gene co-expression network analysis, the essential transcription factors that putatively regulate the biosynthesis of serotonins were revealed. Among those, PnWRKY19-3 was functionally tested and shown to increase the serotonin content in transgenic rice by upregulating *OsT5H* under low temperature conditions. These findings suggest that PnWRKY19-3 may play a positive role in promoting melanin formation in the culms of *P. nigra*. According to the two functional genomic platforms, it appears that low temperature stimulates melanin formation in *P. nigra* by inducing the biosynthesis of indoles. Our research provides new insights into melanin biosynthesis in bamboo, which may be vital to other plant species.

## Introduction

Melanin is a large family of organized biomolecules that can be oligomeric or polymeric in nature and are associated with the pigments typically found in plants, animals, and microorganisms^[1]^. Melanin provides many essential biological functions, including protecting organisms from ultraviolet (UV) damage, endowing them with color, providing a means for temperature regulation and mimetic camouflage, as well as antibacterial and antitumor activity^[1−4]^. There are five types of melanin, including eumelanin and pheomelanin found in animals, allomelanin in plants, neuromelanin and pyomelanin in microorganisms^[5]^. Both eumelanin and pheomelanin are derivatives of tyrosine, while the allomelanin in plants originates from phenolics^[6,7]^. For example, melanin in the seeds of black oats was identified to be a homopolymer of coumaric acids^[8]^, while in *Zanthoxylum bungeanum,* the melanin was shown to contain catechin, hydroquinone, and *p*-coumarin^[9]^. Allomelanin primarily exists in fruit peels or seeds^[9−12]^. It is a class of nitrogen-free polymers, believed to be formed through oxidative polymerization by the oxidative enzymes peroxidase (POD) and polyphenol oxidase (PPO)^[6]^. Furthermore, some plants are also capable of producing phenomelanin or eumelanin. For example, the products of catechol, tyrosine, and some indole derivatives were identified in *Echinacea purpurea*, suggesting it can synthesize the eumelanin and allomelanin^[13]^. In rice (*Oryza sativa*), the the increased accumulation of serotonin has been associated with a dark brown phenotype and stunted growth^[14,15]^. Compared with artificially synthesized melanin, plant melanin is less toxic and widely used in food, medicine, and coloring^[6,16,17]^, all which confer benefits to the human body. As a consequence, the demand for natural plant-derived melanin is increasing exponentially.

The formation of melanin in plants has been shown to be influenced by environmental factors. For instance, the black color common to barley kernel (*Hordeum vulgare*) is considered an adaptive trait for survival in harsh conditions, including cold temperatures, drought, and growth at high altitudes^[18]^. Exposure to high light intensity can inhibit PPO activity, resulting in a decrease in melanin production in the skin of persimmon fruit^[19]^. Moreover, the brown pigmentation in seed coats is known to be induced by low temperatures in soybean (*Glycine max*)^[20]^. Cold storage can increase membranes' permeability and/or disrupt cell compartmentation, causing phenolic compounds to migrate from the vacuoles to the cytosol^[21]^. These phenolics are then oxidized by polyphenol oxidases active in the cytosol, resulting in browning in mung bean (*Vigna radiata*) tissues^[21]^. Cold storage has also been shown to reduce browning, decrease enzymatic activity, and inhibit lignification in post-harvest bamboo shoots^[22]^, but the impact of low temperature on melanin formation in bamboo remains unclear.

In general, most bamboo species have green leaves and culms, and some common varieties have leaves or culms that vary in color from green to yellow, such as *Phyllostachys violascens* cv. Viridisulcat^[23]^ and *P. violascens* var. Flavistriatus^[24]^. Abnormal chloroplast development, decreased chlorophyll, and increased flavonoids are the main reasons that cause the yellowing variation^[23−25]^. Variaties with red or black color are very unique in bamboo. *Indosasa hispida* cv. 'Rainbow' exhibits purplish red at different stages of its growth. Its pigment was ascertained as anthocyanins in previously research, and some genes involved in anthocyanin biosynthesis were also revealed^[26,27]^. *P. nigra* is renowned for the distinctive black coloration of its mature culms and it has been widely utilized in classical landscape architecture. It exhibits natural variation of the green-culmed *P. nigra* var. henonis. Interestingly, the young shoots of both species are green when they sprout, but after about 1 year of development, the culms of *P. nigra* turn completely black, while those of *P. nigra* var. henonis remain green. Cai et al. believe that the black pigments of *P. nigra* culms are also anthocyanins, but most secondary metabolites in the anthocyanin metabolic pathway were not significantly enriched^[28]^. Our preliminary research revealed that the pigment in *P. nigra* is insoluble in distilled water and is primarily soluble in alkaline solutions. The reuslts of a stability analysis showed that it exhibits good light and reduction resistance, as well as iron ion chelation ability^[29]^. Furthermore, the reaction results with 10% sulfuric acid and magnesium powder, as well as hydrochloric acid, were both negative, indicating that the composition of *P. nigra* pigments was not anthocyanins^[29]^. Thus, the composition of melanin in *P. nigra* remains controversial, and its synthesis mechanism remains unclear to date.

In the current study, the physiological indicators, major metabolites, and transcriptomes of *P. nigra* culms were comprehensively examined in attempts to elucidate the melanin composition and the impact of low temperature on its biosynthesis. It appears that the black pigments in the epidermis and cortex of *P. nigra* culms belong to typical melanin. Furthermore, low temperature treatment significantly promotes melanization by mainly enhancing the content of indoles (e.g., serotonin). Several transcription factors (TFs) potentially involved in the formation of melanin were also revealed by weighted gene co-expression network analysis (WGCNA). Among those identified, *PnWRKY19-3* was functionally tested by overexpression in rice and shown to increase serotonin content. These findings implicate *PnWRKY19-3* in regulating serotonin biosynthesis under low temperature conditions, and could be key to promoting the deposition of melanin in *P. nigra* culms.

## Materials and methods

### Plant materials and treatments

For detecting coloration and pigment composition analysis, the culm epidermises were collected from *P. nigra* (Pn) and *P. nigra* var. henonis (Pnh) specimens at Zhejiang Agriculture and Forestry University, China. The culm epidermises were scraped off the bamboo at different development stages: I (the culms of Pn and Pnh were young and green), II (some black spots appeared on the culms of Pn), III (80% culms of the Pn had turned black), and IV (the culms of Pn were totally black). At least five individual bamboo plants each of Pn and Pnh were collected. After collection, the samples were kept in a freezer at −80 °C.

For the low temperature treatment, rhizomes from field-grown Pn were transplanted to pots and cultured in a greenhouse for approximately two years. The immature culms began displaying a full green color in April and continued until June, when the low-temperature treatments were initiated. The greenhouse-grown plants were placed in growth chambers and grown simultaneously at 4 °C (designated as the low temperature treatment, LT) and 25 °C (which served as the experimental control, designated RT for 'room temperature'). A minimum of five young bamboo shoots were employed in each treatment as biological replicates and maintained for 25 d. The culm tissue of the parallel-grown Pn was sampled at 0 h (control), 3, and 24 h and 5, 10, 15, and 25 d following induction at 4 and 25 °C. Following collection, all samples were immediately flash-frozen using liquid nitrogen and stored in a freezer at −80 °C.

### Pigment analysis

For the anthocyanin analysis, about 0.1 g each of the Pn and Pnh samples was extracted using 1 mL of an acidic ethanol solution. After sufficient homogenization, the samples were extracted at 75 °C for 20 min, then the samples were centrifuged at room temperature for 10 min, and the supernatant was collected for testing with a spectrophotometer at 520 nm. The content of anthocyanins was calculated according to the formulas provided in the kit protocol (Keming Biotechnology Co., Ltd., Suzhou, China).

The carotenoid (Car) and chlorophyll (Chl) of Pn and Pnh were extracted using 1 mL of acetone. After extraction, the filters were determined using a spectrophotometer at 645 and 663 nm for Chl, and 470 nm for Car. Then the contents of Chl and Car were estimated using the formulas provided by the associated kits (Keming Biotechnology Co., Ltd., Suzhou, China).

For flavonoids, approximately 0.1 g of the sample was extracted using a 1 mL 60% ethanol solution for 2 h at 60 °C. Then the extraction was centrifuged at room temperature for 10 min at 10,000 × *g*. The supernatant was measured using a spectrophotometer at 510 nm, and the content of flavonoids was calculated using a kit protocol (Keming Biotechnology Co., Ltd., Suzhou, China).

### Tissue sectioning and staining

The culms of Stage IV Pn and Pnh were vertically sliced via an automatic rotary slicer (Leica RM2265, Germany). Before staining, the tissues were trimmed to 50-μm-thick sections. Schmorl staining and Masson–Fontana staining were performed according to the protocols with some modifications (Beijing Leagene Biotechnology Co.,Ltd, China). For Schmorl staining, the sections were stained for 1 min and washed with 1% acetic acid for 1 min. Then the sections were stained with a nuclear counterstaining solution for 3 min and washed with double-distilled water (ddH_2_O) three times before imaging. For Masson–Fontana staining, the sections were stained successively with Fontana silver ammonia solution for 1 min, Haibo solution for 1 min, and a nuclear red staining solution for 1 min. Before imaging, the sections were washed three times with ddH_2_O.

### Extraction and analysis of melanin

The melanin of Pn was isolated with an alkali solution and acid isolation method. Approximately 5 g of PnIV powder was soaked in 50 mL of 1 mol/L NaOH (at a mass/volume ratio of 1:10). The suspension was then hot-soaked in a water bath at 95 °C for 1 h. After extraction, the suspension was centrifuged at 10,000 rpm for 10 min, and the supernatant was retained. The extraction process was repeated twice, and the supernatants were pooled and centrifuged again at 10,000 rpm for 10 min. Finally, the supernatant was adjusted to a pH of 2.0 with a 6 M hydrochloric acid solution and allowed to stand at room temperature for 12 h to precipitate the melanin. The pellet was washed with ddH_2_O until it reached neutrality and then sequentially washed three times with chloroform, ethanol, and ethyl acetate to remove impurities. The final precipitate was vacuum-dried and stored at −20 °C.

The solubility of the melanin extracts was tested according to a previous study^[19]^. Here, ddH_2_O (pH 6.8), NaOH (pH 8.0), and six organic solvents, namely chloroform, ethanol, ethyl acetate, acetone, acetonitrile, and dimethyl sulfoxide (DMSO) were used^[19]^. In each test, about 1 mg of the melanin sample was mixed with 1 mL of the solutions above, and the mixture was incubated at 25 °C for 4 h. Then the solubility of melanin was determined after the mixture was centrifuged at 10,000 rpm for 5 min. For precipitation and oxidation measurements, FeCl_3_ (10%, 125 μL), HCl (3 mol/L, 80 μL), and H_2_O_2_ (30%, 125 μL) were added to the melanin solutions (1 mg/mL)^[19]^. The results were analyzed after incubating the mixture for 30 min before centrifugation at 10,000 rpm for 5 min.

### Ultraviolet–visible absorption and Fourier transform infrared spectroscopy

The extracted melanin was dissolved in a 0.5 mol/L NaOH solution (10 mg/L) and used to detect absorbance using a UV-26001 spectrophotometer (Shimadzu, Japan) in the range of 200−700 nm. As a control, 0.5 mol/L NaOH was used.

About 10 mg of the isolated melanin was mixed with KBr and used for Fourier transform infrared (FT-IR) spectroscopy using a Thermo Nicolet IS 5 (Thermo Scientific, USA) equipped with a diamond Attenuated Total Reflectance (ATR) accessory. The measurement was performed in the range of 4,000−600 cm^−1^ with an optical resolution of 4 cm^−1^.

### Radical scavenging activity

The 2,2-diphenyl-1-picrylhydrazyl (DPPH) and 2,2-azino-bis-3-ethylbenzothiazoline-6-sulphonic acid (ABTS) radical scavenging activities were determined using kits from Solarbio Science & Technology Co., Ltd. (Beijing, China). Briefly, 20 mg fresh epidemic samples of the Pn and Pnh culms at Stage IV were extracted with 1 mL ofthe extraction solution and incubated for 30 min at 40 °C. After centrifuging for 10 min at 10,000 rpm, the supernatant was used to measure the DPPH and ABTS using a spectrophotometer at 515 and 405 nm, respectively. The DPPH and ABTS values were calculated using the formulas provided in the protocols.

### Color parameters

To record the change in color, the brightness (ΔL) and chromaticity indices (Δa and Δb) were measured using a CR-10 portable colorimeter (Konica Minolta, Japan). The ΔL value represents the difference in brightness, where positive values indicate white tones and negative values represent dark tones. The Δa value represents the red and green spectrum, where positive values indicate a reddish color and negative values indicate a greenish color. The Δb value represents the yellow and blue spectrum, where positive values indicate a yellowish color and negative values indicate a bluish color. For each biological specimen, grown at both temperatures, at least 10 values were recorded for each time point.

### RNA sequencing

The total RNA of stored, flash-frozen culm specimens of *P. nigra* (CK, RT1d, LT1d, RT15d, LT15d, RT25d, and LT25d) was extracted using a Plant RNA Extraction Kit (Takara, Japan). The purity of the total RNA was analyzed using an Agilent Bioanalyzer 2100 (Agilent, CA, USA). Only RNA samples that demonstrated structural integrity with a 260/280 nm absorbance ratio between 1.8 and 2.0 and an RNA integrity number (RIN) of ≥ 7.0 were used to generate cDNA libraries. Following quality confirmation and mRNA isolation, paired-end sequencing was performed via the Illumina Novaseq TM 6000 sequence platform. For data analysis, clean reads were mapped to the reference genome of *P. edulis* (version 2018)^[30]^ using HISAT2 (https://daehwankimlab.github.io/hisat2/, version: hisat2-2.2.1), and mapped reads were assembled using StringTie (http://ccb.jhu.edu/software/stringtie/, version: stringtie-2.1.6). The FPKM (fragments per kilobase of transcript per million mapped reads) value of all transcripts was calculated by StringTie and ballgown (www.bioconductor.org/packages/release/bioc/html/ballgown.html). Principal component analysis (PCA) was performed using R (www.r-project.org) to reveal the relationships among the samples. For the differentially expressed gene (DEG) analysis, DESeq2 software was employed, and only genes with a fold change ≥ 2 and a false discovery rate (FDR) ≤ 0.05 were considered to be DEGs. Finally, the DEGs were subjected to Gene Ontology (GO) database and Kyoto Encyclopedia of Genes and Genetics (KEGG) pathway enrichment analyses^[31]^.

### Metabolomic profiling analysis

The metabolites were extracted from the culm epidermis of *P. nigra* (CK, RT1d, LT1d, RT10d, LT10d, RT15d, LT15d, RT25d, and LT25d). A Vanquish Flex HPLC system (Thermo Fisher Scientific, Bremen, Germany) fitted with an ACQUITY UPLC T3 column (100 mm × 2.1 mm, 1.8 μm, Waters, Milford, USA) maintained at 35 °C was used for chromatographic separation, using the following parameters. The flow rate was maintained 0.4 mL/min, and the mobile phase comprised Solvent A (water, 0.1% formic acid) and Solvent B (acetonitrile, 0.1% formic acid). The gradient elution conditions were as follows: 0 to 0.5 min, 5% B; 0.5 to 7 min, 5% to 100% B; 7 to 8 min, 100% B; 8 to 8.1 min, 100% to 5% B; 8.1 to 10 min 5% B. A high-resolution tandem mass spectrometer (Q-Exactive, Thermo Scientific) was used to detect metabolites, which was operated in both positive and negative ion modes. Specifically, precursor spectra were collected at a resolution of 70,000 to achieve an automatic gain control (AGC) target of 3 e^6^. The maximum injection time was set to 100 ms. A Top 3 configuration was established to acquire data in data-dependent acquisition (DDA) mode. Fragment spectra were collected at a resolution of 17,500 to reach an AGC target of 1 e^5^, with a maximum injection time of 80 ms. To assess the stability of the liquid chromatography-mass spectrometry (LC-MS) throughout the entire acquisition, a quality control sample (a pool of all samples) was collected after every 10 samples. The raw data files were processed by the XCMS, CAMERA, and metaX toolbox, implemented with R software, and then the online KEGG database and Human Metabolome Database (HMDB) were used to annotate the metabolites. The normalized data were subjected to PCA analysis to evaluate their quality. Student's *t*-test was used to detect significant differentially accumulating metabolites (DEMs), and the metabolites between two samples with a matched ratio of ≥ 2 or ≤ 1/2; *p*-value < 0.05 and VIP (variable important for the projection) ≥ 1 were considered to be DEMs. Finally, the annotation of DEMs was performed using the online KEGG database.

### WGCNA analysis

To analyze the correlation between the major DEMs and the DEGs, a WGCNA was performed (www.omicstudio.cn/analysis/wgcna?id=16). The gene number (minimum size) was 30, and the minimum height for merging modules was set to 0.25. After obtaining highly related genes, a correlation analysis was then performed using the online COR tool (www.omicstudio.cn/tool/62). To generate gene networks, only highly correlated genes (|cor| > 0.99, *p* < 0.01) were used, and Cytoscape (3.9.1) was used for visualization.

### Gene cloning and overexpression

The coding sequence (CDS) of *PnWRKY19-3* was determined on the basis of transcriptome alignment and named according to what has been described previously^[32]^. The primers used for cloning are shown in Supplementary Table S1. The CDS of *PnWRKY19-3* was inserted into a pCAMBIA:1300 vector, which was driven by the *CaMV 35S* promoter and had the *GFP* gene added at the 3' nopaline synthase (NOS) terminator (35S::PnWRKY19-3-GFP). The construct was then introduced into rice via *Agrobacterium tumefaciens* (EHA105)-mediated transformation following a previously described method^[33]^. Positive transformants were confirmed by screening genomic DNA isolated from the flag leaves using a cetrimonium bromide (CTAB) method. Following confirmation, the overexpressed (OE) and wild-type (WT) lines were cultivated in half-strength Hoagland solution in a greenhouse, and two-month-old plants were subsequently used for the RT and LT treatments. The experimental design with the transgenic rice mirrored the experimental design of the RT and LT treatment of bamboo, lasting for approximately 30 d. For each treatment, six WT plants and five OE plants were employed, and the samples were collected at 0 and 3 h and 1 and 5 d following temperature treatment for subsequent determination of the serotonin content and gene expression analysis.

### Subcellular localization

The 35S::PnWRKY19-3-GFP construct was also used for subcellular localization in the transient expression system of tobacco leaves^[34]^; the empty pCAMBIA1300-GFP vector was used as a negative control. After 3 d of postinfiltration, the transfected leaves were observed under a confocal microscope (ZEISS, LSM 880). Green fluorescent protein (GFP) fluorescence was detected under a 488-nm argon laser.

### Serotonin measurement

The serotonin content in WT and *PnWRKY19-3* OE lines of rice was measured as previously described^[35]^. Briefly, after collection, the flag leaves (0.1 g) were ground with 1.2 mL of methanol and immediately mixed for 5 min at 4 °C. The supernatant was collected by centrifugation at 13,500 × *g* for 5 min at 4 °C and transferred to a new tube using a syringe fitted with a 0.22 μm filter. The filtered solution was slowly injected into a preactivated Sep-Pak C18 cartridge, and the cartridge was washed with 1.5 mL (the same volume as the sample) of 80% methanol. The washing solutions were combined with the filtrate, and the mixture was evaporated to dryness and dissolved in 0.2 mL of 50% methanol. This solution was analyzed by high-performance liquid chromatography (HPLC) (Agilent 1260 Infinity II) using a Sepax Bio-C18 column (4.6 mm × 250 mm) with an isocratic elution of 5% (v/v) methanol in water containing 0.3% trifluoroacetic acid at a flow rate of 0.8 mL/min. The eluents were detected at a wavelength of 280 nm on a diode array detector (DAD). The quantification was conducted with three independent extractions.

### Transient overexpression in protoplasts

The CDS of *PnWRKY19-3* was connected to the vector of pUBQ10 as described in a previous study^[25]^. The protoplasts were isolated from tender branches of *P. nigra* using a polyethylene glycol (PEG)-mediated method^[36]^. Then the recombinant vector was transiently overexpressed in the protoplast of *P. nigra*^[25]^. The cells were collected after overnight cultivation.

### Quantitative reverse transcription–polymerase chain reaction analysis

Total RNA from the flag leaves of WT and *PnWRKY19-3* OE rice, RNA from the culm epidermis of Pn and Pnh under LT and RT treatments, and RNA from the *PnWRKY19-3* OE protoplasts was extracted using TRIzol reagent. Then the single-stranded DNA was synthesized using the PrimeScript™ RT reagent kit with gDNA Eraser (TaKaRa). Quantitative reverse transcription–polymerase chain reaction (qRT-PCR) was performed using SYBR Green Master Mix (Bio-Rad) on a real-time polymerase chain reaction (PCR) instrument (Bio-Rad). The reaction system was as follows: 5 μL SYBR Gr, 0.5 μL forward primer, 0.5 μL reverse primer, 2 μL cDNA, and 3 μL H_2_O. The reaction program was as follows: denaturation at 95 °C for 30 s, annealing at 60 °C for 30 s, and extension at 72 °C for 30 s, repeated for 40 cycles. The *Ubiquitin* genes in rice (*OsUBQ*) and in bamboo (*PeUBQ*) were used as reference genes^[37,38]^, and the relative expression of the transgene was calculated using the 2^−^^ΔΔ^^Cᴛ ^method. The formula for ΔΔCᴛ was: ΔΔCᴛ = ΔCᴛ (target genes) − ΔCᴛ (reference gene). Finally, the primers used for qRT-PCR are shown in Supplementary Table S1.

### Statistical analysis

The data were analyzed by IBM SPSS V27.0, using one-way analysis of variance (ANOVA) and Student's *t*-test. Means ± standard deviations (SDs) are presented, calculated from at least three independent experiments. Statistical significance was assumed when *p* < 0.01.

## Results

### Morphological observation and pigment analysis of Pn and Pnh culms

**Figure 1:**
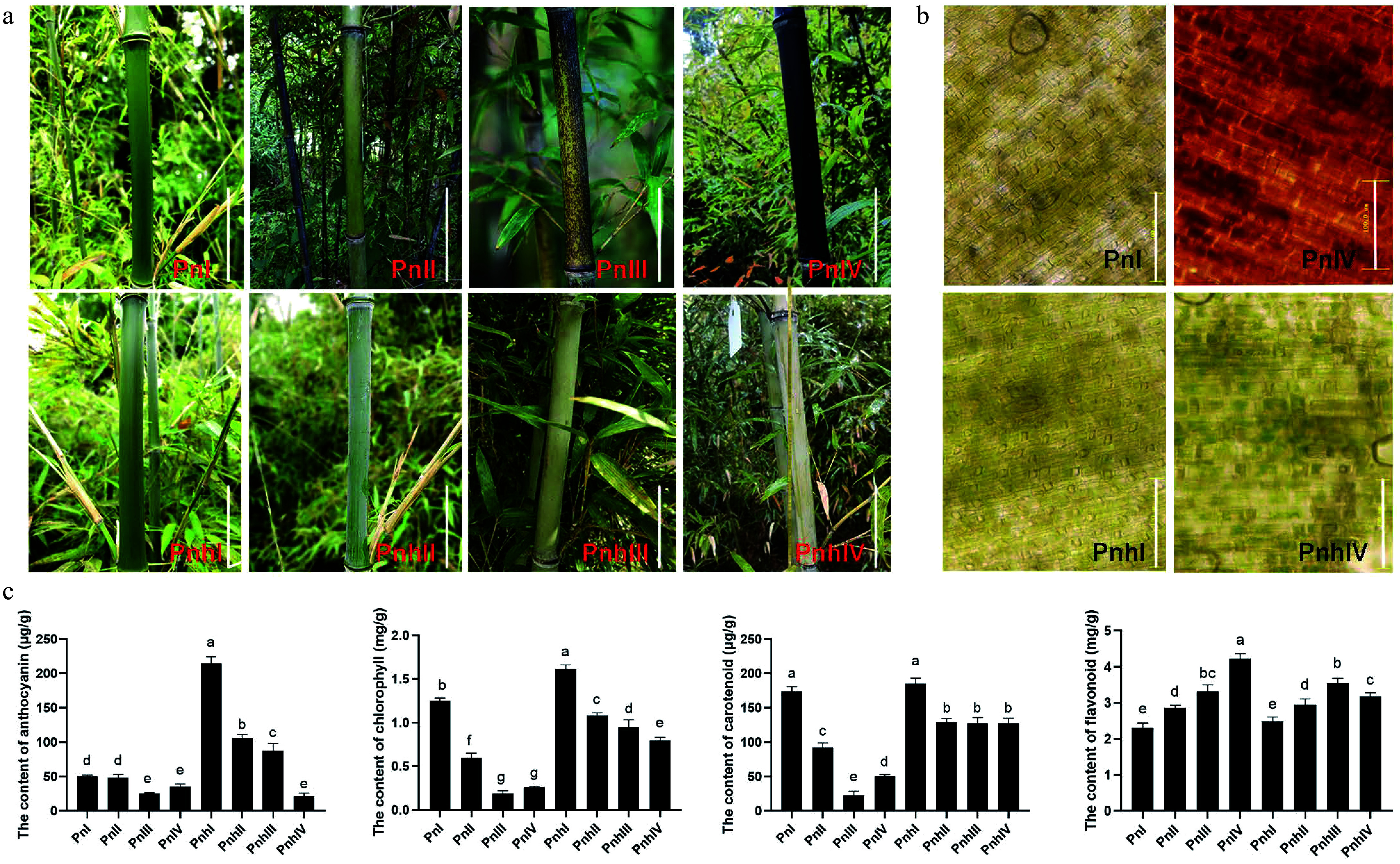
Morphological observation and pigment analysis in Pn and Pnh culms. (a) The coloration process of Pn and Pnh at different stages. Bar = 10 cm. (b) Observation of pigments in Pn and Pnh at Stages I and IV. Bar = 100 μM. (c) Analysis of the content of anthocyanins, chlorophyll, carotenoids, and flavonoids in Pn and Pnh at various stages. Different letters indicate significant differences in the data.

The coloration process of Pn and Pnh culms was observed in this study. They were all green when immature (PnI and PnhI, Fig. 1a). For Pn, the black spots began to appear on the culms at Stage II. These spots significantly accumulated during Stage III, resulting in completely purple-black culms when matured (Stage IV). However, the culms of Pnh remained green at all stages (Fig. 1a). Furthermore, changes in the pigments of Pn and Pnh culms were observed. As shown in Fig. 1b, the epidermal cells of Pn were filled with green chlorophyll (Chl) during the Stage I. However, the Chl degraded and the purple-black pigments significantly accumulated at Stage IV. For Pnh, the cells were full of Chl regardless of the stages. This suggests that the enriched black pigments led to a variation in color in the Pn culms. To reveal the composition of black pigments, the content of anthocyanins, Chl, Car, and flavonoids was measured in the culm epidermis of Pn and Pnh (Fig. 1c). Anthocyanin, Chl, and Car all showed a decreasing trend in Pn during its coloration, and their levels were significantly lower than those in Pnh. In contrast, the content of total flavonoids increased in Pn, and only in the last stage was it significantly higher than in Pnh bamboo. In summary, the anthocyanins, carotenoids, and flavonoids were not the main pigments responsible for the black phenotype of Pn culms.

### Analysis of melanin from Pn culms

**Figure 2:**
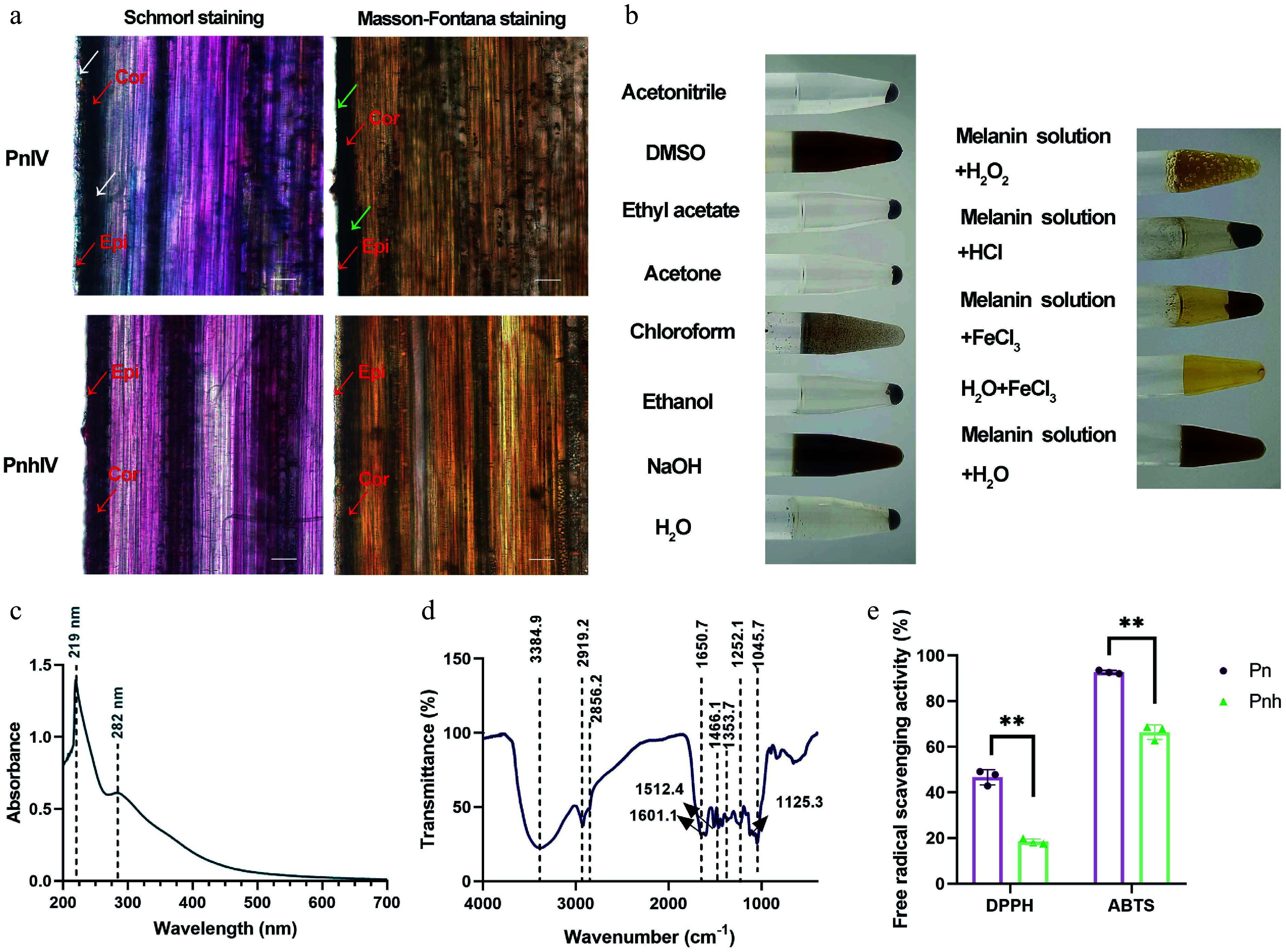
The physical and chemical properties analysis of the melanin isolated from Pn. (a) The tissue sectioning and staining of Pn and Pnh culms. Red arrows: Cor, cortex; Epi, epidermis. White arrows: blue signals after staining via Schmorl's method. Green arrows: black signals after Masson–Fortuna staining. Bar = 50 μm. (b) The solubility, precipitation, and oxidation of the melanin isolated from Pn. (c), (d) The UV and FT-IR analysis of Pn melanin. The dashed lines represent the absorption peaks. (e) The DPPH and ABTS free radical scavenging analysis of Pn and Pnh at Stage IV. ** represents *p* < 0.01.

To identify whether the pigment of Pn culms belongs to melanin, culms of PnIV and PnhIV were stained using the Schmorl and Masson–Fortuna methods. As shown in Fig. 2a, the epidermis and cortex of Pn appeared blue under Schmorl staining, but those of Pnh showed no signals. Moreover, the epidermis and cortex of Pn appeared black under Masson–Fortuna staining, while those of Pnh were brown. Then the pigments of Pn and Pnh were extracted using an alkali solution and acid isolation method. As a result, the pigments in Pn were precipitated as melanin, but those of Pnh were not (Supplementary Fig. S1). The isolated melanin of Pn was completely dissolved by NaOH and partially dissolved by DMSO, but was insoluble in water and other organic solvents, such as ethanol, acetone, ethyl acetate, and acetonitrile (Fig. 2b). Moreover, it was precipitated by FeCl_3_ and HCl, and lightened by the H_2_O_2_ solution (Fig. 2b). The UV-visible spectrum of the isolated melanin exhibited two maximum absorption peaks at 219 and 282 nm (Fig. 2c). Additionally, the absorption bands of the isolated melanin appeared a 2,800−3,400 cm^−1^ and 1,050−1,650 cm^−1^ (Fig. 2d). Finally, the radical scavenging activity of Pn and Pnh was measured. Compared with Pnh, the DPPH and ABTS values in Pn were both significantly higher, indicating a greater antioxidant activity (Fig. 2e). Taken together, the results of the analyses of physical and chemical properties indicate that the primary pigment in the epidermis and cortex of Pn belongs to plant melanin, which is responsible for the black phenotype of its culm.

### LT accelerates melanin formation in Pn culms

**Figure 3:**
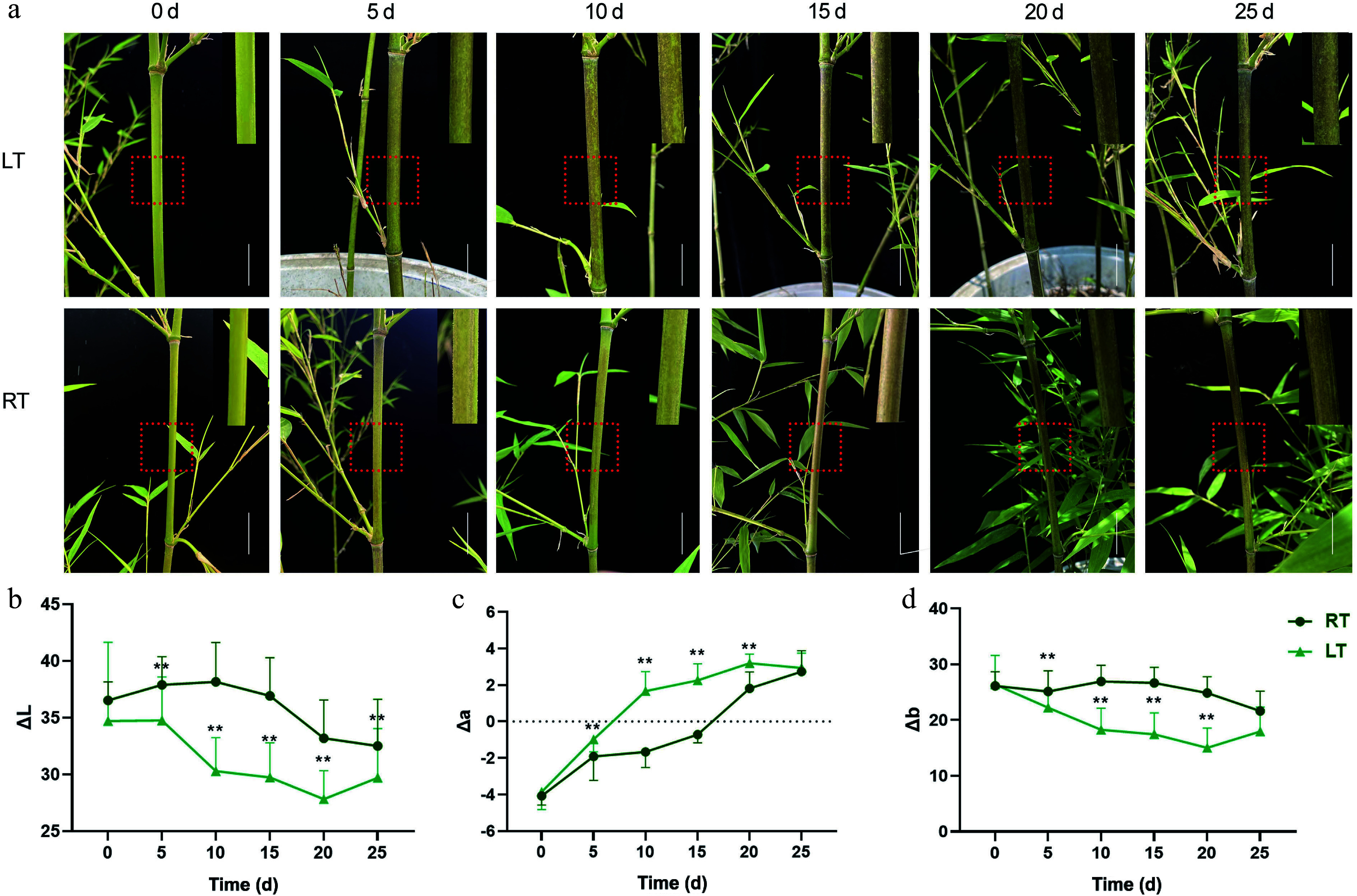
Growth phenotype and color difference of Pn grown under the low temperature (LT) and room temperature (RT) treatments. Bar = 2 cm. The red dotted box was enlarged in the upper right corner of each image. (a) The phenotype of Pn grown under the LT and RT treatments over the duration of the 25-day temperature trial. (b−d) Changes in ΔL, Δa, and Δb values of Pn grown under the LT and RT treatments. ΔL represents the brightness, Δa represents the color change from red to green, and Δb represents the color change from yellow to blue. ** represents *p* < 0.01.

Three-month-old greenhouse-propagated Pn plants were subjected to LT and RT treatments for 25 d. Throughout the 25-day trial, the color of the culms in both treatment groups gradually changed from green to black (Fig. 3a). Under RT, a few black spots appeared after the fifth day, and by Day 15, the spots had become increasingly noticeable. At the end of the trial, approximately 80% of the Pn culms had turned black. In contrast, the culms grown at LT showed distinct black spots on the fifth day. By Day 10, significantly more spots emerged, resulting in almost completely black culms by the 25^th^ day. To further analyze the changes in culm color, the optical color parameters ΔL, Δa, and Δb were measured (Fig. 3b–d). For the RT treatment, both the ΔL and Δb values initially increased but then decreased, while the Δa values increased and exceeded 0 by Day 20. In contrast, when grown at LT, both the ΔL and Δb values initially decreased before increasing, and Δa increased significantly, exceeding zero by Day 10. Moreover, the ΔL and Δb values of the LT-grown bamboo were significantly lower than those grown at RT, while the Δa value was significantly higher. In summary, the LT treatment significantly accelerated blackening of the Pn culms.

### LT induces the accumulation of indole-derived compounds in the culms of Pn

**Figure 4:**
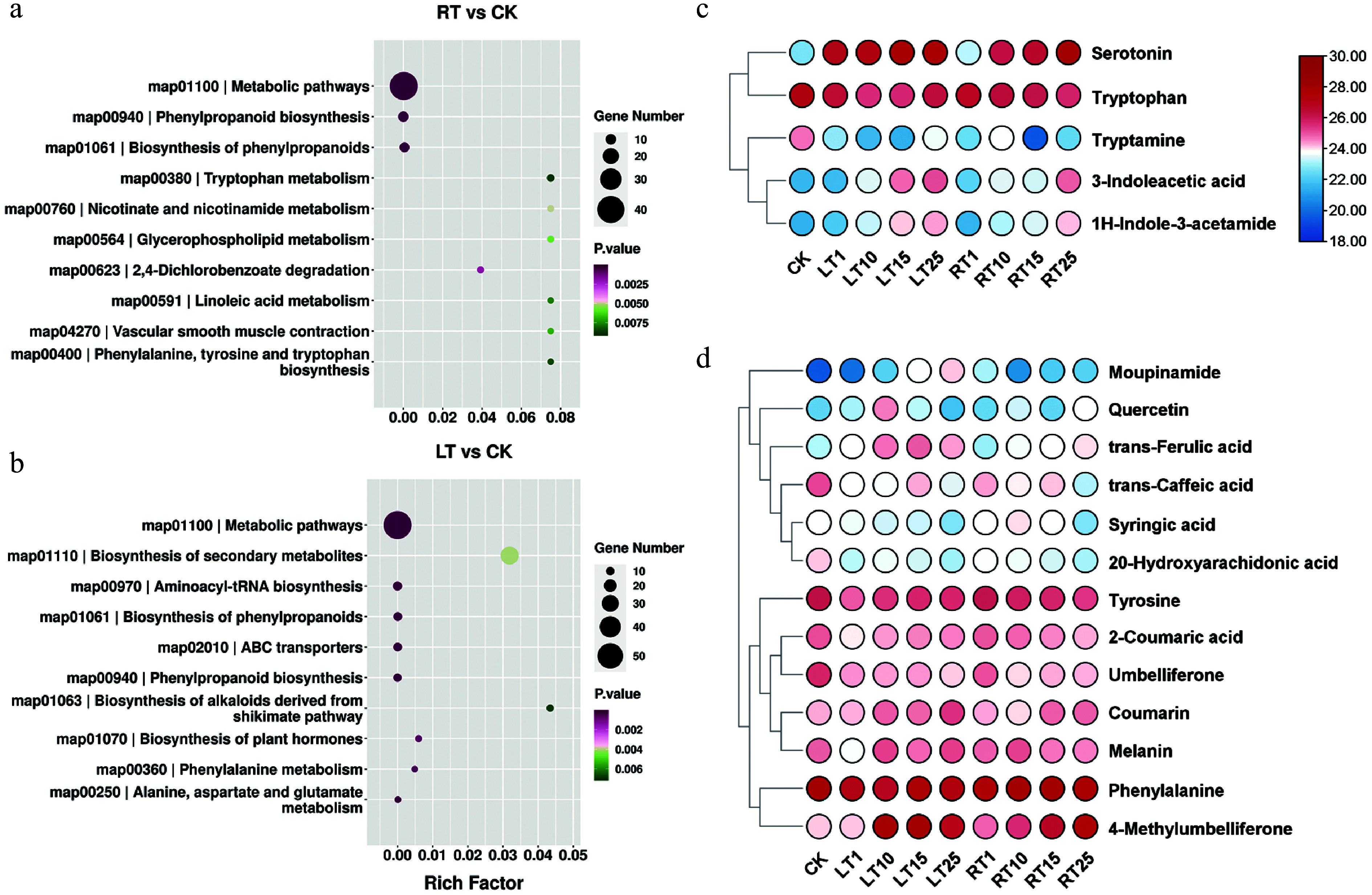
Metabolic profiles of Pn grown under LT and RT. KEGG enrichment analysis of DEMs under the (a) RT treatment and (b) LT treatment. (c) Heatmap highlighting the relative contents of indole-related metabolites when grown under the LT and RT treatments. (d) Heatmap highlighting the relative contents of phenolic-related metabolites when grown under the LT and RT treatments.

To investigate the changes in melanin-associated metabolites under LT, a nontargeted metabolic profiling method was employed. According to the PCA results, the different biological replicates of individuals grown at the same temperature and at the same time points grouped well, and most samples from the RT treatment were separated from the LT treatments, indicating that the experiment was reproducible and generally reliable (Supplementary Fig. S2). Compared with RT, 4,115 and 4,803 DEMs were identified after Days 1 and 15 of LT treatment, respectively; on Day 25, the number of DEMs increased to 5,283 (Supplementary Table S2). For both LT and RT, the DEMs that accumulated largely belonged to the core phenylpropanoid biosynthetic pathway derived from phenylalanine metabolism. However, the biosynthesis of alkaloids derived from shikimate biosynthesis; plant hormone biosynthesis; and the alanine, aspartate, and glutamate metabolism pathways were also found to be enriched only under the LT treatment (Fig. 4a, b). Metabolites involved in indole synthesis and phenolic metabolism were the primarily focus, as a consequence of their essential roles in forming melanin in plants. As shown in Fig. 4c, the abundance of indole derivatives, including serotonin, 3-indoleacetic acid, and 1H-indole-3-acetamide, significantly increased during LT treatment compared with the RT treatment. However, the profiles of tryptamine and tryptophan (Trp) showed no significant difference between LT and RT. Compared with the indoles, most phenolics exhibited comparable levels between treatments; only trans-ferulic acid (FA), 4-methylumbelliferone (4-MU), and coumarin were shown to significantly accumulate during LT (Fig. 4d). These findings indicate that LT may stimulate the accumulation of indoles and their derivatives, which may result in the accumulation of melanin in the culms of *P. nigra*.

### Identification of DEGs during LT treatment by transcriptome analysis

**Figure 5:**
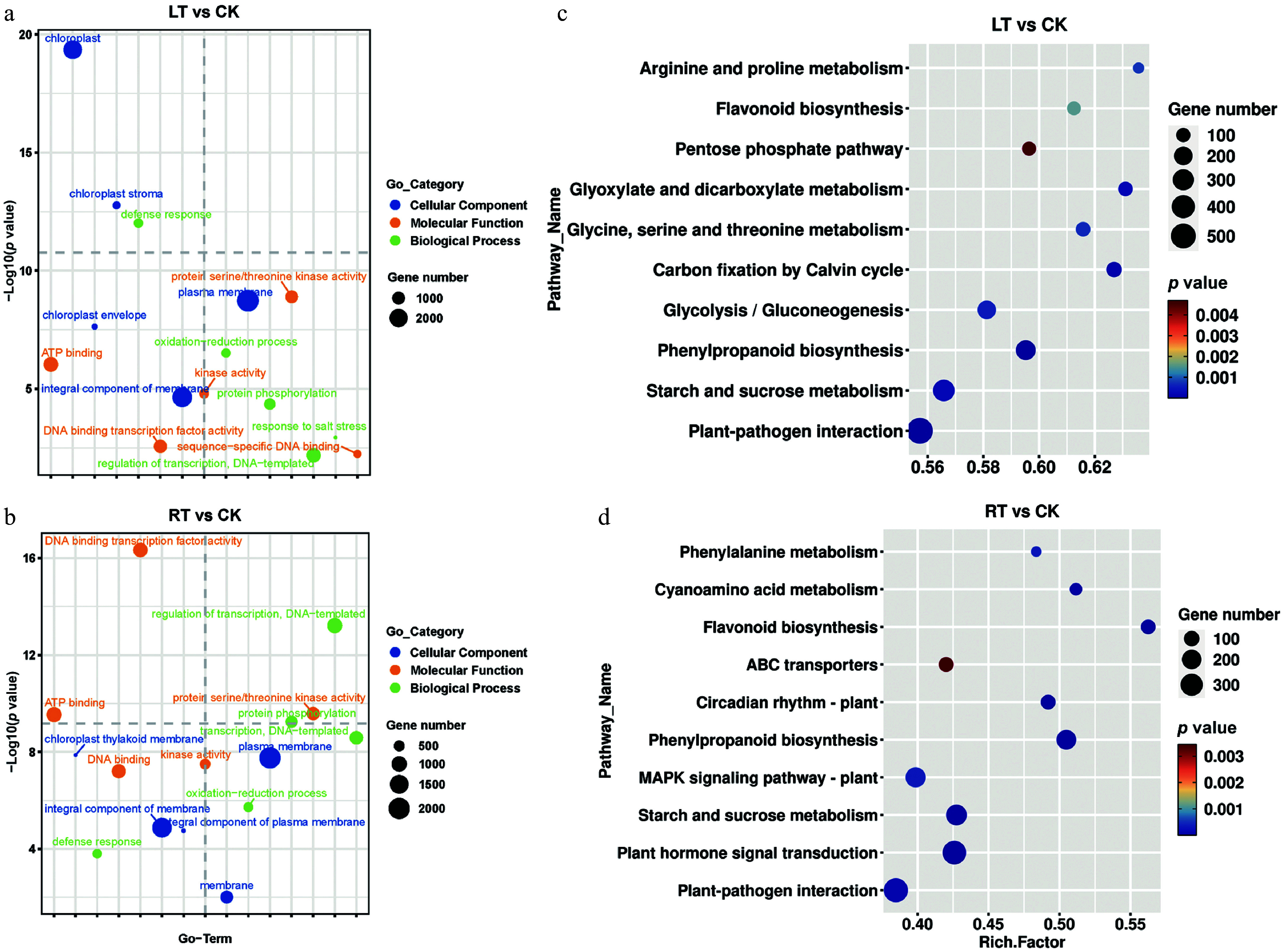
GO terms and KEGG enrichment analysis of DEGs in *P. nigra* comparing the RT and LT growth treatments. GO enrichment analysis of DEGs under (a) the LT treatment and (b) the RT treatment. KEGG enrichment analysis of DEGs under (c) the LT treatment and (d) the RT treatment.

To better understand the molecular mechanism underpinning melanin formation during the LT treatment, transcriptome sequencing was performed. More than 5.5 GB of clean data was obtained in each sample, with the G20 and G30 values exceeding 99% and 96%, respectively. The alignment rate for all samples was over 90%, and the GC content was approximately 50% (Supplementary Table S3). According to the PCA analysis, there was a very strong correlation among biological repeats under both treatment conditions, supporting the reliability of the RNA-seq data (Supplementary Fig. S3). A total of 5,025, 5,357, and 4,150 upregulated genes and 10,258, 10,063, and 5,004 downregulated genes were identified when comparing LT1 vs RT1, LT15 vs RT15, and LT25 vs RT25, respectively (Supplementary Fig. S3). The GO and KEGG analyses revealed that the DEGs that responded to the LT and RT treatments were primarily associated with the plasma membrane, integral membrane components, and adenosine triphosphate (ATP) binding, and were mainly involved in pathways related to plant–pathogen interactions, starch and sucrose metabolism, phenylpropanoid biosynthesis, and the flavonoid biosynthesis pathway (Fig. 5a, b). Additionally, the DEGs highlighted by the LT condition were specifically related to the chloroplast, chloroplast stroma and the chloroplast envelope, and accumulated in the pathways of carbon fixation by the Calvin cycle; glycine, serine and threonine metabolism; and glycolysis/gluconeogenesis (Fig. 5c, d).

### Identification of DEGs involved in the biosynthesis of indole and its derivatives in Pn under the LT treatment

**Figure 6:**
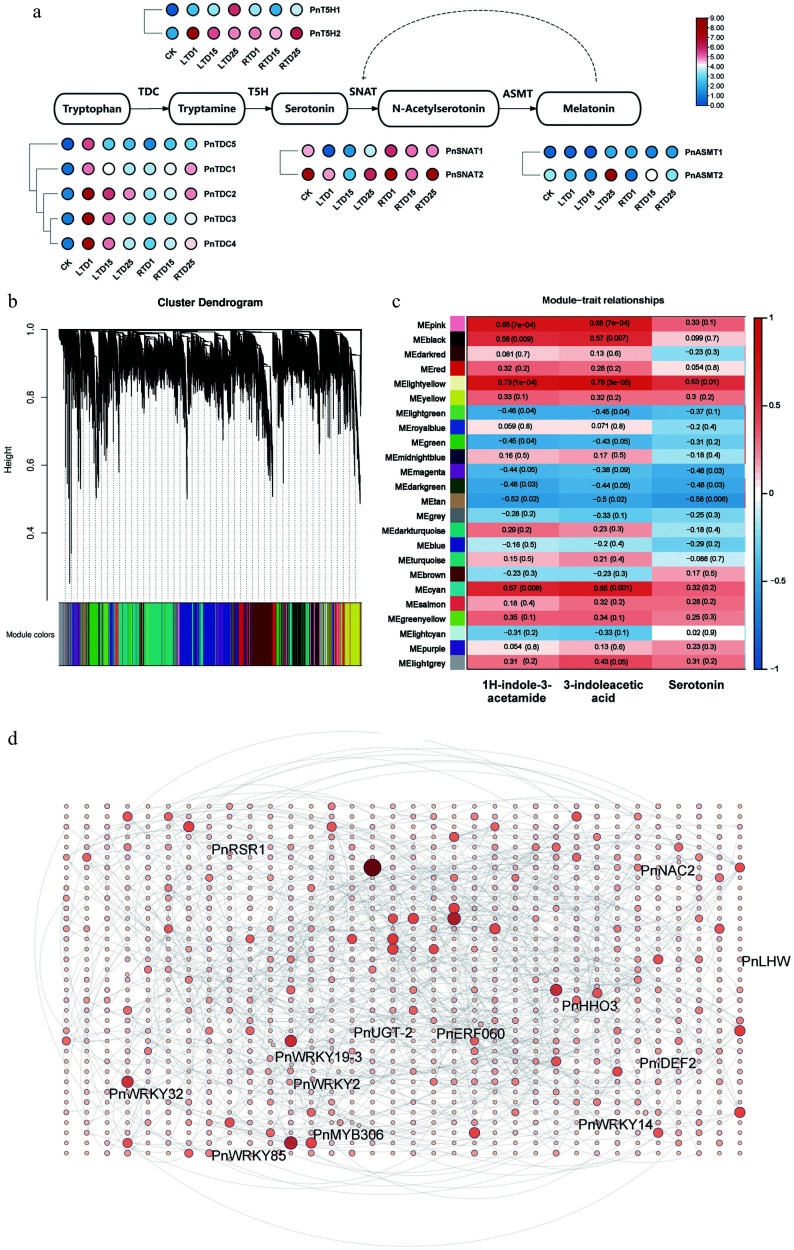
Correlation analysis and expression profiles of DEGs related to indole biosynthesis. (a) Heatmap highlighting the expression profiles of genes involved in indole biosynthesis. (b) Division of genes and TFs into 24 modules. (c) Correlation analysis of gene co-expression network modules with serotonin, 3-indoleacetic acid, and 1H-indole-3-acetamide. (d) Gene network analysis of the aforementioned genes that are highly related to serotonin, 3-indoleacetic acid, and 1H-indole-3-acetamide. The red circles represent various genes, and the shades and sizes indicate the number of gene connections.

As the derivatives of indoles (serotonin, 3-indoleacetic acid, and 1H-indole-3-acetamide) were significantly enriched under the LT treatment, the expressions of genes involved in the Trp biosynthesis pathway were analyzed in this study. Specifically, *PnTDCs*, *PnT5Hs*, and *PnASMT-2*, which are known to be part of the pathway, were significantly upregulated by the LT treatment, whereas *PnSNATs* was downregulated (Fig. 6a). WGCNA was performed to identify the co-expressed gene modules. After filtering, the genes were divided into 24 modules according to their expression patterns (Fig. 6b). Serotonin, 3-indoleacetic acid, and 1H-indole-3-acetamide were then selected for correlation analysis with the 24 modules (Fig. 6c). The heatmap of module–trait correlations indicated that the genes in the pink, cyan, and light yellow modules had a strong positive correlation with both 3-indoleacetic acid and 1H-indole-3-acetamide. In contrast, only the light yellow genes exhibited a relatively high positive correlation with serotonin (Fig. 6c). A total of 1,190 annotated genes and TFs were identified from these three modules, including 6 WRKYs (WRKYGQK), 8 MYBs (v-myb avian myeloblastosis viral oncogene homolog), and 3 NACs (NAM, ATAF1/2, CUC1/2). To identify the hub TFs involved in the biosynthesis of serotonin, 3-indoleacetic acid, and 1H-indole-3-acetamide, a gene network was generated (Fig. 6d). The result indicated that *PnWRKY19-3*, *PnHHB5*, *PnERF060*, and *PnNAC2* had more than 10 targeting relationships, suggesting an essential role in regulating the biosynthesis of indoles in the culms of Pn when grown under the LT treatment. Finally, the expression levels of *PnTDCs*, *PnT5Hs*, *PnWRKY19-3* were verified using qRT-PCR (Supplementary Fig. S4). As a result, they showed similar expression patterns to those in RNA-seq, suggesting the reliability of our RNA-seq data.

### *PnWRKY19-3* enhances the content of serotonin in transgenic rice

**Figure 7:**
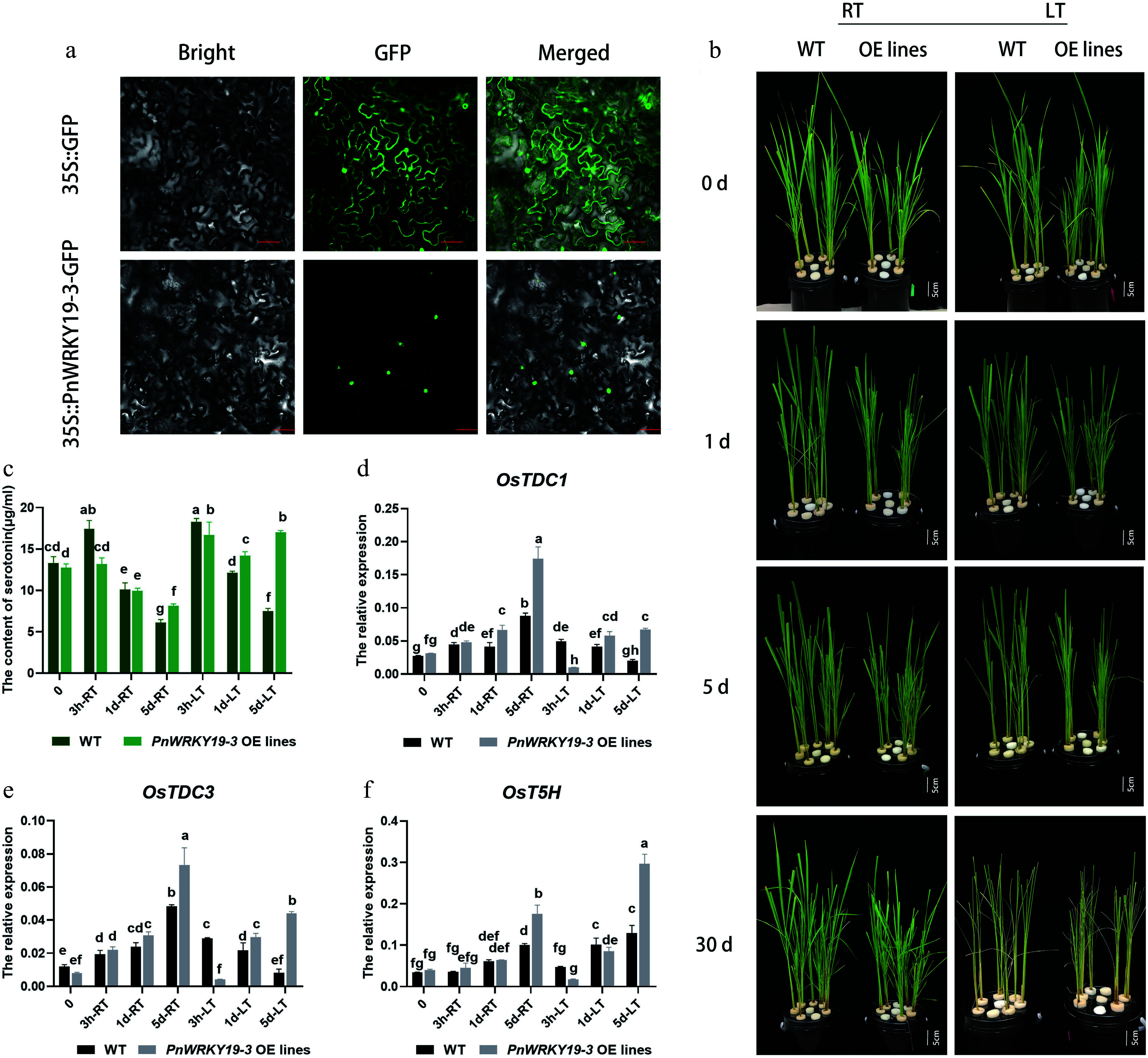
*PnWRKY19-3* overexpression can enhance the accumulation of serotonin in rice. (a) Nuclear localization of PnWRKY19-3 protein; bar = 50 μM. (b) Phenotypes of the WT and *PnWRKY19-3* overexpressing transgenic rice grown under RT and LT conditions; bar = 5 cm. (c) Serotonin content in WT and *PnWRKY19-3* overexpressing transgenic rice when grown at under RT and LT conditions. (d–f) Expression analysis of *OsTDC1*, *OsTDC3*, and *OsT5H* in WT and *PnWRKY19-3* overexpressing transgenic rice grown under RT and LT conditions. Different letters indicate significant differences in the data.

In plants, the WRKY family serves as the core regulatory hub of secondary metabolic networks involved in the biosynthesis pathways of flavonoids, carotenoids, and anthocyanins, as well as the Trp-derived secondary metabolic pathway^[39]^. In this study, *PnWRKY19-3* showed a strong correlation with serotonin in the culms of Pn under the LT growth treatment and was therefore selected for in-depth functional testing *in planta*. According to our study, PnWRKY19-3-GFP was only detected in the nucleus of tobacco cells (Fig. 7a), indicating nuclear localization. Fourteen transgenic lines of rice overexpressing *PnWRKY19-3* were created (Supplementary Fig. S5) and grown under both RT or LT conditions (Fig. 7b). Under the RT treatment, *PnWRKY19-3* OE rice exhibited normal growth phenotypes similar to the WT, where both showed leaf yellowing after 5 d of growth under LT conditions and withering after 30 d of treatment. *PnWRKY19-3* did not appear to affect the development and LT response of the transgenic rice plants (Fig. 7b). Serotonin content was measured in both WT and *PnWRKY19-3* OE plants, where it was shown that the serotonin in WT plants initially increased and then decreased under both the RT and LT treatments. In contrast, in *PnWRKY19-3* OE plants, serotonin exhibited a similar trend to WT under RT conditions, but when grown at LT, serotonin levels increased significantly and surpassed those in the WT plants after 1and 5 d of the LT treatment (Fig. 7c). The expression of key genes involved in serotonin biosynthesis was also analyzed. In WT plants, the expression levels of *OsTDC1* and *OsTDC3* increased under RT conditions, but decreased under the LT treatments. In *PnWRKY19-3* OE plants, *OsTDC1* and *OsTDC3* exhibited similar expression patterns to WT plants grown at RT; however, under LT conditions, both genes showed an increasing trend, and their expression levels were significantly higher than those of WT plants. Despite this increase, their expressions were lower compared with the RT condition (Fig. 7d, e). The transcript abundance of *OsT5H* was elevated in both WT and *PnWRKY19-3* OE plants under both RT and LT conditions. Notably, compared with the WT, the expression of *OsT5H* was significantly higher in *PnWRKY19-3* OE plants when grown under LT conditions (Fig. 7f). In summary, *PnWRKY19-3* appears to enhance serotonin production in transgenic rice, primarily by upregulating *OsT5H*. *PnWRKY19-3* was transiently overexpressed in the protoplasts of *P. nigra* (Supplementary Fig. S6). In *PnWRKY19-3* OE cells, *PnT5H* was slightly induced. It is hypothesized that *PnWRKY19-3* might indirectly upregulate *PnT5H* in *P. nigra*.

## Discussion

Currently, bamboo forests cover about 20 million hectares worldwide. Bamboo is widely distributed in many countries, including China and India in the Asia-Pacific, Guatemala and Columbia in the Americas, Kenya and Ethiopia in Africa, with significant stands in Mexico. Most bamboo species are predominantly green, although some varieties exhibit green with yellow stripes, yellow with green stripes, or a more yellowed appearance^[23−25]^. The reduction in green color is primarily attributed to abnormal chloroplasts and decreased chlorophyll levels^[24,25]^. Additionally, the presence of enriched flavonoids contributes to the yellow phenotype^[23,24]^. Some highly distinctive bamboo varieties inherently possess high ornamental and cultural value because of their reddish or purplish hues^[26,27,40]^. The purplish-red pigment found in 'Rainbow' bamboo has been attributed to anthocyanins, which include compounds such as cyanidin-3-O-glucoside chloride, pelargonin chloride, and delphinidin chloride^[26]^. The genes associated with anthocyanin biosynthesis in rainbow bamboo have been revealed and described^[27]^. The culms of *P. nigra* exhibit colors similar to those of rainbow bamboo; however, the nature of the pigment is different and has been shown to contain double bonds and a phenolic hydroxyl structure, indicating the presence of polyphenolic compounds rather than anthocyanins^[29]^. Previous studies have also shown that *P. nigra* has inherently high concentrations of total phenolics in its shoots^[41]^, but the specific phenolic substances and the precursors responsible for the unique coloration have yet to be identified.

In the present study, the primary pigments responsible for the black phenotype in *P. nigra* were identified to be melanin. Moreover, *P. nigra* was treated at different temperatures to examine the effect of temperature on inducing melanization. Consequently, LT was found to have a positive impact on accelerating melanin formation, primarily by enhancing the accumulation of indoles and their derivatives. A series of key biosynthetic genes was additionally shown to be significantly induced by the LT treatment, including *PnTDC* and *PnT5H*, which are involved in serotonin biosynthesis. Moreover, several TFs were also identified to have high positive correlations with indoles under the LT treatment by WGCNA. Among those identified, *PnWRKY19-3* was functionally tested by creating rice overexpressing the gene, where the resulting plants showed a higher serotonin content relative to WT plants grown under similar conditions, implicating this TF in melanin biosynthesis under LT. These findings have provided new insights into the complex networks that govern melanization in *P. nigra*.

### The black pigments in the *P. nigra* culms are melanin

Plant melanin can be divided into anthocyanin melanin and 'allomelanin'^[6]^. Unlike anthocyanin melanin, allomelanin is generally soluble in alkaline solutions and insoluble in water and most organic solvents, and generally has strong physicochemical properties such as oxidation-reduction, metal ion binding ability, photothermal stability, and photoconductivity^[6]^. In this study, we found that the content of anthocyanins was significantly lower in *P. nigra* compared with its green variety (Fig. 1). Moreover, the black pigments mainly accumulated in the epidermis and cortex of *P. nigra* culms, and appeared blue and black under Schmorl and Masson–Fortuna staining, respectively (Fig. 2a). Schmorl and Masson–Fortuna staining are two common methods used to identify melanin. When stained via either of these two methods, melanin will turn blue and black becasue of its high reducibility^[42]^. Furthermore, the extracted pigments of *P. nigra* were found to be completely dissolved by NaOH but remained insoluble in water and most organic solvents. Additionally, they could be precipitated with FeCl_3_ and HCl and lightened with H_2_O_2_ (Fig. 2b). These findings are consistent with characteristics of melanin in mushrooms, persimmon, and other plants^[19,43,44]^. As in previous studies, natural melanin exhibits high antioxidant activity^[1,45−47]^. For instance, the free radical scavenging rates were increased with increased melanin contents in three *Auricularia heimuer* specimens^[47]^, and the DPPH, ABTS, and ferric reducing ability of plasma (FRAP) values in black proso millet were significantly higher than those in other colored varieties^[45]^. Here, the DPPH and ABTS values in Pn at Stage IV were significantly higher than those in Pnh at Stage IV, which corresponds to the characteristics of a typical melanin, as shown in other plants^[45−47]^. Additionally, Song et al. found that eumelanin can produce a maximum absorbance peak at 220 nm, while allomelanin often exhibits the highest absorption value at 200–300 nm and displays a typical small protein absorption peak at 270−280 nm. In the FT-IR spectra, the typical absorption bands of eumelanin were at 3,500−3,000 cm^−1^ (stretching vibrations of O-H in carboxyl, phenols, and -NH groups), 1,650−1,500 cm^−1^ (C=C, COO), and 1,400−1,300 cm^−1^ (C-H, C-O, C-C), 1,710 (C=O); the broad band of allomelanin was between 1,200 and 1,400 cm^−1^ (C-O)^[5]^. Herein, the maximum absorption peaks at 219 and 282 nm in the UV region and the FT-IR absorbance peaks at about 3,384 and 1,600−1,000 cm^−1^ reflect a putative mixed melanin in *P. nigra.* Similar results were also detected in *E. purpurea*, which are capable of synthesizing pheomelanin and eumelanin^[13]^. Taken together, *P. nigra* may also have the ability to produce different types of melanin.

### LT plays a positive role in accelerating melanin formation in *P. nigra* culms

Temperature is a key environmental factor impacting plant development. It is well known, for example, that it can influence pigmentation by changing the contents of anthocyanins or flavonoids in many plants^[48−50]^. For example, LT has been shown to positively affect melanin formation in soybean and barley^[18,20]^. Similarly, the primary substrates of polyphenol oxidase in mung bean have been shown to increase during cold storage, while the activity of PPO itself remains unaffected^[21,51]^. Here, the contents of the indoles (e.g. serotonin, 3-indoleacetic acid, and 1H-indole-3-acetamide) were significantly increased by the LT treatment (Figs 4 & 5), implying that LT likely also promotes the formation of melanin in *P. nigra* culms by enhancing the concentration of available precursors for oxidation. According to Kogo et al., cold storage may also cause an increase in membrane permeability or disrupted cell compartmentation, resulting in phenolic substrates eluting from the vacuoles to the cytosol. These phenolics can then be oxidized by the existing localized PPOs active in the cytosol, leading to tissue browning^[21]^. Therefore, LT treatment may also cause a cell rupture in *P. nigra* culms, but this remains unresolved and requires further investigation.

### LT can enhance serotonin accumulation in *P. nigra* culms

Indole and its derivatives, such as serotonin and other 5-hydroxy indoles, can serve as substrates for phenoloxidases to produce indole-melanin^[52,53]^. Serotonin is a precursor for several secondary metabolites, including indole glucosinolates, phytoalexins, and alkaloids. Additionally, serotonin has antioxidant activity and contributes to the maintenance of free radical homeostasis within cells^[54−56]^. In rice, the accumulation of serotonin manifests in a dark brown appearance in the endosperm or panicles^[14]^, and increased levels of serotonin and its precursors can also cause a dark brown pigmentation in rice leaves^[15]^. Herein, serotonin and its derivatives accumulated under LT, suggesting it may be involved directly in the formation of melanin in *P. nigra* culms. In plants, serotonin originates from Trp, with L-tryptophan decarboxylases (TDC) and tryptamine 5-hydroxylase (T5H) being the primary limiting enzymes in its biosynthesis, where serotonin N-acetyltransferase (SNAT) and acetylserotonin O-methyltransferase (ASMT) then convert serotonin to melatonin through a series of reactions^[57−59]^. Environmental and internal factors can regulate serotonin biosynthesis by activating these biosynthetic genes. For example, *OsbZIP18* was induced by UV-B stress, leading to activation of the serotonin biosynthesis genes *OsTDC1*, *OsTDC3*, and *OST5H*, and consequently producing serotonin in rice^[15]^. Moreover, in banana, *MaTDC* and *MaASMT* were shown to be upregulated by LT stress^[59]^. In this study, *PnTDC* and *PnT5H* were significantly upregulated by LT, highlighting their importance in promoting serotonin biosynthesis in *P. nigra*. Furthermore, several TFs (i.e., PnWRKY19-3 and PnNAC2) were also highly correlated with serotonin formation. In addition, we found that overexpressing *PnWRKY19-3* in rice can enhance serotonin accumulation in transgenic lines, primarily by upregulating *OsT5H* under LT (Fig. 7). It can also slightly induce *PnT5H* in the overexpressed protoplast of *P. nigra*. Li et al., on the basis of phylogenetic analysis, showed that *PnWRKY19-3* was homologous to *OsWRKY19* and belonged to the WRKY Group III subfamily^[32]^. The WRKY family serves as the core regulatory hub of secondary metabolic networks in plants and is involved in the biosynthesis pathways of flavonoids, carotenoids, and anthocyanins, as well as the Trp-derived secondary metabolic pathway^[39]^. In *Arabidopsis thaliana*, the WRKYs regulate the Trp-derived metabolic pathway mainly through AtWRKY33, which can interact with ERF1 to induce camalexin biosynthesis^[60]^. In rice, *OsWRKY14* was clearly shown to be involved in serotonin biosynthesis by binding to the W-box elements of the TDC1 promoter^[61]^. In cassava, MeWRKY20/75 was shown to form a protein complex with MeTDC2 and MeASMT2/3 to participate in Trp biosynthesis^[62]^. Recently, OsWRKY19 was shown to increase the level of a pyrrole, thereby improving the aroma in rice^[63]^. Pyrrole is a key substrate for the synthesis of indoles. Collectively, we hypothesized that PnWRKY19-3 may be involved in melanin formation by upregulating serotonin biosynthesis in *P. nigra* under LT growth conditions*.*

## Conclusions

The culms of *P. nigra* exhibit a distinct black color due to the accumulation of melanin. Our results suggest that LT can accelerate melanin production by increasing the synthesis of indoles and their derivatives (serotonin, 3-indoleacetic acid, and 1H-indole-3-acetamide). Several genes directly involved in the biosynthesis of serotonins, including *PnTDC* and *PnT5H*, were found to be significantly upregulated by the LT treatment, highlighting their importance in melanin formation. Additionally, the overexpression of a cold-induced gene, *PnWRKY19-3*, can enhance serotonin content in rice by upregulating *OsT5H* under LT conditions, indirectly implicating this transcription factor in serotonin biosynthesis in *P. nigra*. Overall, LT appears to promote melanin production in *P. nigra* culms by increasing the level of serotonin, likely through the upregulation of *PnWRKY19-3* and its target genes.

## SUPPLEMENTARY DATA

Supplementary data to this article can be found online.

## Data Availability

The RNA-seq data were deposited in the GEO database of NCBI with the accession number GSE254522.
