# Supplementary material for: Integrative transcriptome and metabolome evaluation of melanin biosynthesis in Phyllostachys nigra during low-temperature growth
Source: For Res (Fayettev). 2025 Sep 23;5:e020. doi: 10.48130/forres-0025-0020 (PMC12464485; doi:10.48130/forres-0025-0020)
Supplement: Supplementary file 1 — Supplementary data to this article can be found online. [file forres-0025-0020-Supplementary.zip › 10.48130_forres-0025-0020-Suppl-FigureS3.pdf]

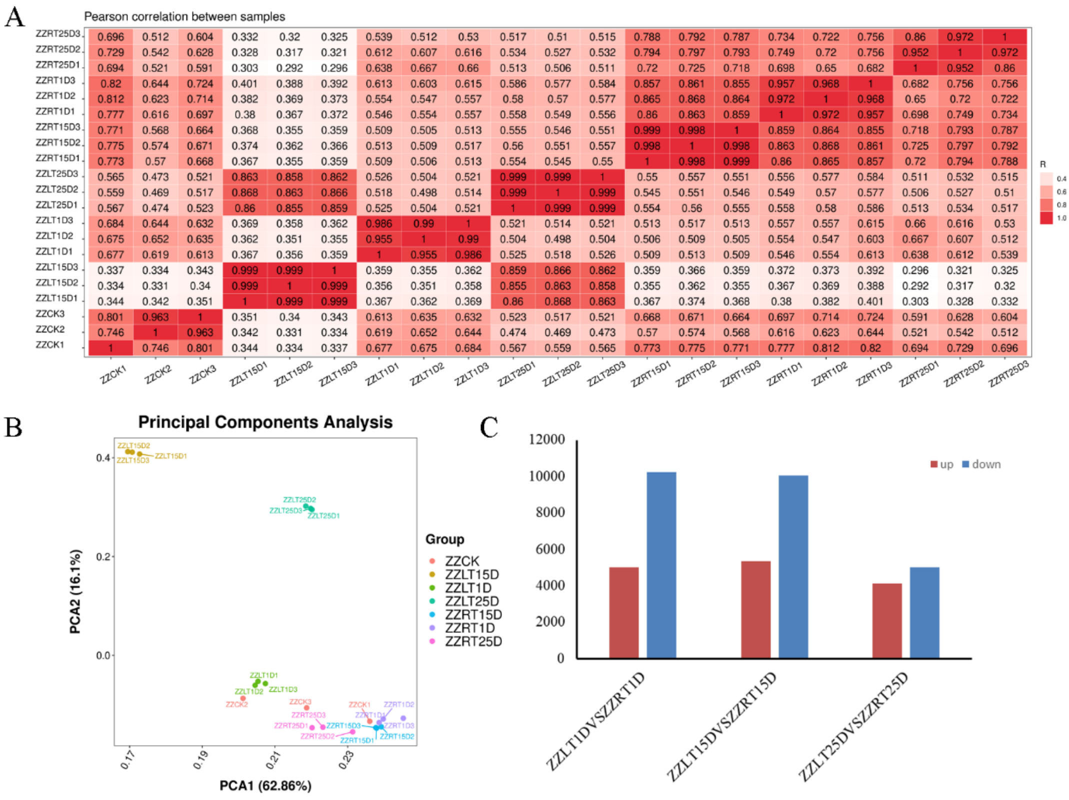

Figure S3 (A) Analysis of RNA-seq data, (B) PCA analysis of RNA-seq data, and (C) Number of DEGs in different comparisons.
