# Supplementary material for: Integrative transcriptome and metabolome evaluation of melanin biosynthesis in Phyllostachys nigra during low-temperature growth
Source: For Res (Fayettev). 2025 Sep 23;5:e020. doi: 10.48130/forres-0025-0020 (PMC12464485; doi:10.48130/forres-0025-0020)
Supplement: Supplementary file 1 — Supplementary data to this article can be found online. [file forres-0025-0020-Supplementary.zip › 10.48130_forres-0025-0020-Suppl-TableS1.pdf]

Table S1 The primer list

	Primer names	Sequence
For gene cloning	PnWRKY19-3F	ATGGGGAGCAACAACAGGACA
	PnWRKY19-3R	TCAATCGATCCATGTATTATTGCTCGGA
For overexpression & Subcellular location	1300PnWRKY19-3F	gagctcggtacccggggatccATGGGGAGCAACAACAGGACA
	1300PnWRKY19-3R	catgtcgactctagaggatccATCGATCCATGTATTATTGCTCGGA
For qRT-PCR	QPCR-PnWRKY19-3F	CTCCTGCTGCAATCCCAATC
	QPCR-PnWRKY19-3R	TCCATGAGTAGCCATCGTCC
	QPCR-PnTDC1-1F	ACCATGAAGGAGCTCAAGGC
	QPCR-PnTDC1-1R	CTTCGTTGGATCGAGACCGT
	QPCR-PnTDC1-2F	GTGCTTCCCAACGTGAAACC
	QPCR-PnTDC1-2R	AGGCCTTGAAGAACGTGGAG
	QPCR-PnTDC1-3F	CTTGAGGTCATGCAGGCTGA
	QPCR-PnTDC1-3R	GCTCCACTTGGCTTGATCCT
	QPCR-PnTDC3-1F	TATCGAGCGTGTTGACTCCA
	QPCR-PnTDC3-1R	TCACGAACATAAAGGCAGGT
	QPCR-PnTDC3-2F	CCCCAACTTCTTCGCCTTCT
	QPCR-PnTDC3-2R	ACGTGGACCCATGCATTGAA
	QPCR-PnTDC3-3F	CCCCAACTTCTTCGCCTTCT
	QPCR-PnTDC3-3R	ACGTGGACCCATGCATTGAA
	QPCR-PnT5H1-1F	CTCTGGAGTGGGTGATGACG
	QPCR-PnT5H1-1R	AAGGTCTCCTTGATGACGGC
	QPCR-PnT5H1-2F	TGACCTCAGCGAGTGTTTCC
	QPCR-PnT5H1-2R	CCTTGATGATGGCACGCATG
	PeUBQ-F	GGGTCGTCCAGTGTCCTCTATTA
	PeUBQ-R	TCAACCAAACCACTGTACCTCAG
	QPR-OsTDC1F	AAGAAACACAAGCCTGCGG
	QPR-OsTDC1R	GAAGCTCTCGTCGATCTGGA
	QPR-OsTDC3F	AGTACATCCTGGAGACGAGC
	QPR-OsTDC3R	GTGTAGACGCCGATCTCCAT
	QPR-OsT5HF	CACAAAGGAGCAGACCAACG
	QPR-OsT5HR	ACGTCGTCATCAGGTTCCAT
	OsUBQF	ACCACTTCGACCGCCACTACT
	OsUBQR	ACGCCTAAGCCTGCTGGTT
