# Supplementary material for: Integrative transcriptome and metabolome evaluation of melanin biosynthesis in Phyllostachys nigra during low-temperature growth
Source: For Res (Fayettev). 2025 Sep 23;5:e020. doi: 10.48130/forres-0025-0020 (PMC12464485; doi:10.48130/forres-0025-0020)
Supplement: Supplementary file 1 — Supplementary data to this article can be found online. [file forres-0025-0020-Supplementary.zip › 10.48130_forres-0025-0020-Suppl-TableS2.pdf]

Table S2 The comparisons of DEMs under LT and RT treatments

comparison	pos_all	pos_up	pos_down	neg_all	neg_up	neg_down	all_regulate
ZZLT1D/ZZCK	2475	789	1686	1996	636	1360	4471
ZZLT15D/ZZCK	2455	1225	1230	1769	970	799	4224
ZZLT25D/ZZCK	3520	1465	2055	2565	1161	1404	6085
ZZLT15D/ZZLT1D	2640	1782	858	2243	1487	756	4883
ZZLT25D/ZZLT1D	3205	1816	1389	2562	1531	1031	5767
ZZLT25D/ZZLT15D	1463	637	826	1140	480	660	2603
ZZRT1D/ZZCK	477	225	252	361	181	180	838
ZZRT15D/ZZCK	2072	910	1162	1448	634	814	3520
ZZRT25D/ZZCK	3498	1524	1974	2481	1125	1356	5979
ZZRT15D/ZZRT1D	1696	842	854	1197	650	547	2893
ZZRT25D/ZZRT1D	3331	1472	1859	2494	1195	1299	5825
ZZRT25D/ZZRT15D	1401	586	815	1063	462	601	2464
ZZLT1D/ZZRT1D	2204	750	1454	1911	683	1228	4115
ZZLT25D/ZZRT25D	2980	1447	1533	2303	1152	1151	5283
ZZLT10D/ZZCK	3450	1418	2032	2524	966	1558	5974
ZZLT10D/ZZLT1D	2634	1546	1088	2068	1229	839	4702
ZZLT15D/ZZLT10D	1386	884	502	1143	706	437	2529
ZZLT25D/ZZLT10D	2637	1324	1313	1957	1053	904	4594
ZZLT10D/ZZRT10D	2672	1104	1568	2131	866	1265	4803
ZZRT10D/ZZCK	1720	771	949	1368	583	785	3088
ZZRT10D/ZZRT1D	1282	669	613	1016	494	522	2298
ZZRT15D/ZZRT10D	925	468	457	624	388	236	1549
ZZRT25D/ZZRT10D	2416	1144	1272	1742	887	855	4158
