# Supplementary material for: Integrative transcriptome and metabolome evaluation of melanin biosynthesis in Phyllostachys nigra during low-temperature growth
Source: For Res (Fayettev). 2025 Sep 23;5:e020. doi: 10.48130/forres-0025-0020 (PMC12464485; doi:10.48130/forres-0025-0020)
Supplement: Supplementary file 1 — Supplementary data to this article can be found online. [file forres-0025-0020-Supplementary.zip › 10.48130_forres-0025-0020-Suppl-TableS3.pdf]

Table S3. The quality analysis of RNA-seq data

Sample	Raw Data Reads	Raw Data bases(G)	Valid Data Reads	Valid Data bases(G)	Valid Ratio(%)	Q20%	Q30%	GC content%
ZZCK1	41547452	6.23G	39661076	5.95G	95.46	99.95	97.66	48.50
ZZCK2	41530548	6.23G	39239078	5.89G	94.48	99.95	97.55	49
ZZCK3	38816196	5.82G	37478582	5.62G	96.55	99.95	97.51	49
ZZLT15D1	45328450	6.80G	42500550	6.38G	93.76	99.94	96.58	50.50
ZZLT15D2	45371568	6.81G	42958952	6.44G	94.68	99.95	96.89	50
ZZLT15D3	42052120	6.31G	39596230	5.94G	94.16	99.94	96.74	50.50
ZZLT1D1	48379840	7.26G	45571744	6.84G	94.20	99.94	97.56	48.50
ZZLT1D2	43617732	6.54G	41496556	6.22G	95.14	99.95	97.53	49.50
ZZLT1D3	42955000	6.44G	40727774	6.11G	94.81	99.95	97.57	49.50
ZZLT25D1	44263874	6.64G	40907008	6.14G	92.42	99.95	97.06	48.50
ZZLT25D2	50035098	7.51G	45742832	6.86G	91.42	99.95	97.07	48.50
ZZLT25D3	46296974	6.94G	42420914	6.36G	91.63	99.95	96.96	49
ZZRT15D1	41605960	6.24G	39740034	5.96G	95.52	99.92	96.48	49.50
ZZRT15D2	40577180	6.09G	38848998	5.83G	95.74	99.94	96.67	49.50
ZZRT15D3	37861972	5.68G	36208014	5.43G	95.63	99.93	96.48	49.50
ZZRT1D1	40069442	6.01G	38538064	5.78G	96.18	99.95	97.49	49
ZZRT1D2	41457996	6.22G	39860590	5.98G	96.15	99.95	97.62	48.50
ZZRT1D3	38900446	5.84G	37549238	5.63G	96.53	99.95	97.62	48.50
ZZRT25D1	44161890	6.62G	41554774	6.23G	94.10	99.95	97.34	48.50
ZZRT25D2	40994848	6.15G	38814202	5.82G	94.68	99.95	97.75	48.50
ZZRT25D3	43649258	6.55G	41200940	6.18G	94.39	99.95	97.32	48.50
