# Supplementary figures and images for: Integrative transcriptome and metabolome evaluation of melanin biosynthesis in Phyllostachys nigra during low-temperature growth
Source: For Res (Fayettev). 2025 Sep 23;5:e020. doi: 10.48130/forres-0025-0020 (PMC12464485; doi:10.48130/forres-0025-0020)

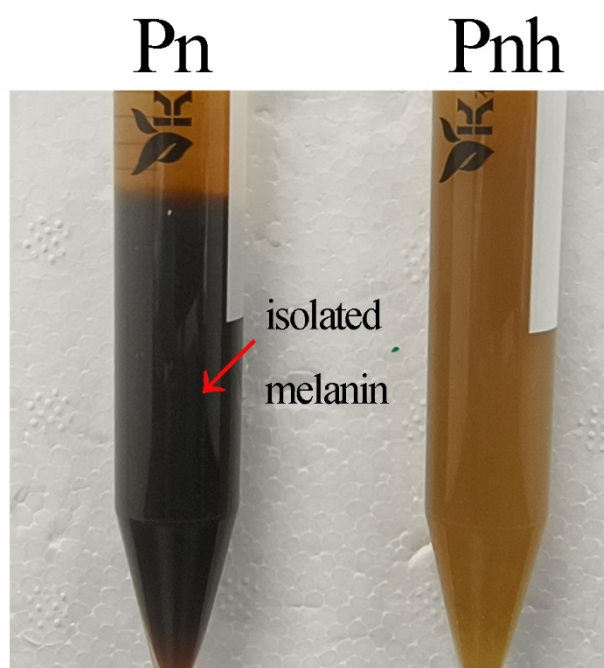

Figure S1. The extraction of the melanin from Pn.

Supplement: Supplementary file 1 — Supplementary data to this article can be found online. [file forres-0025-0020-Supplementary.zip › 10.48130_forres-0025-0020-Suppl-FigureS1.pdf]

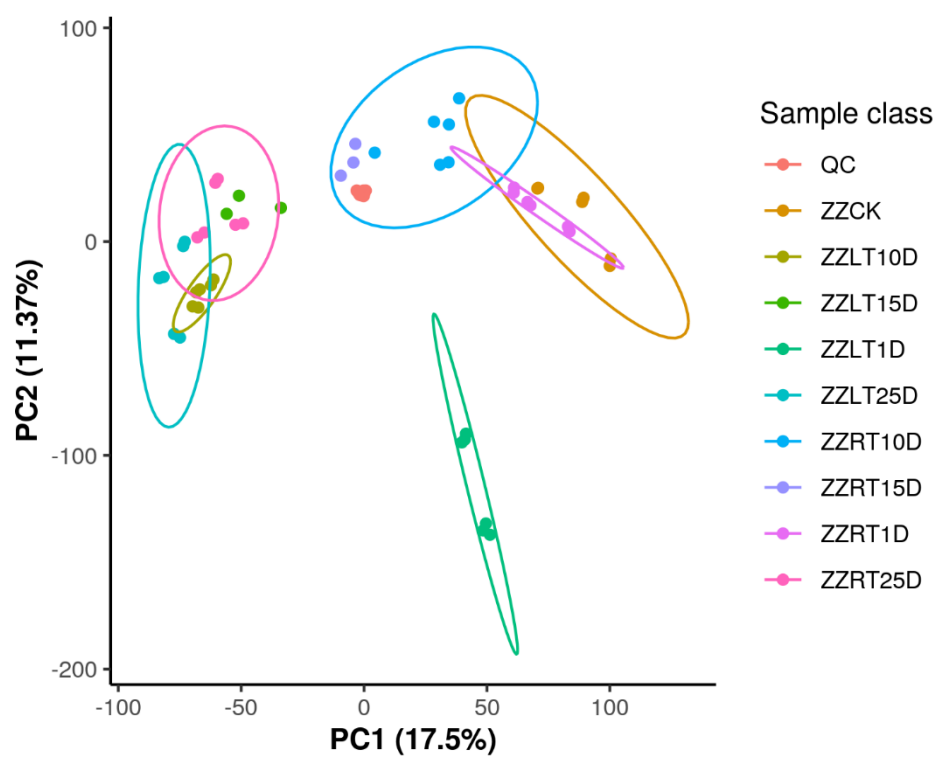

Figure S2. PCA analysis of metabolomic data

Supplement: Supplementary file 1 — Supplementary data to this article can be found online. [file forres-0025-0020-Supplementary.zip › 10.48130_forres-0025-0020-Suppl-FigureS2.pdf]

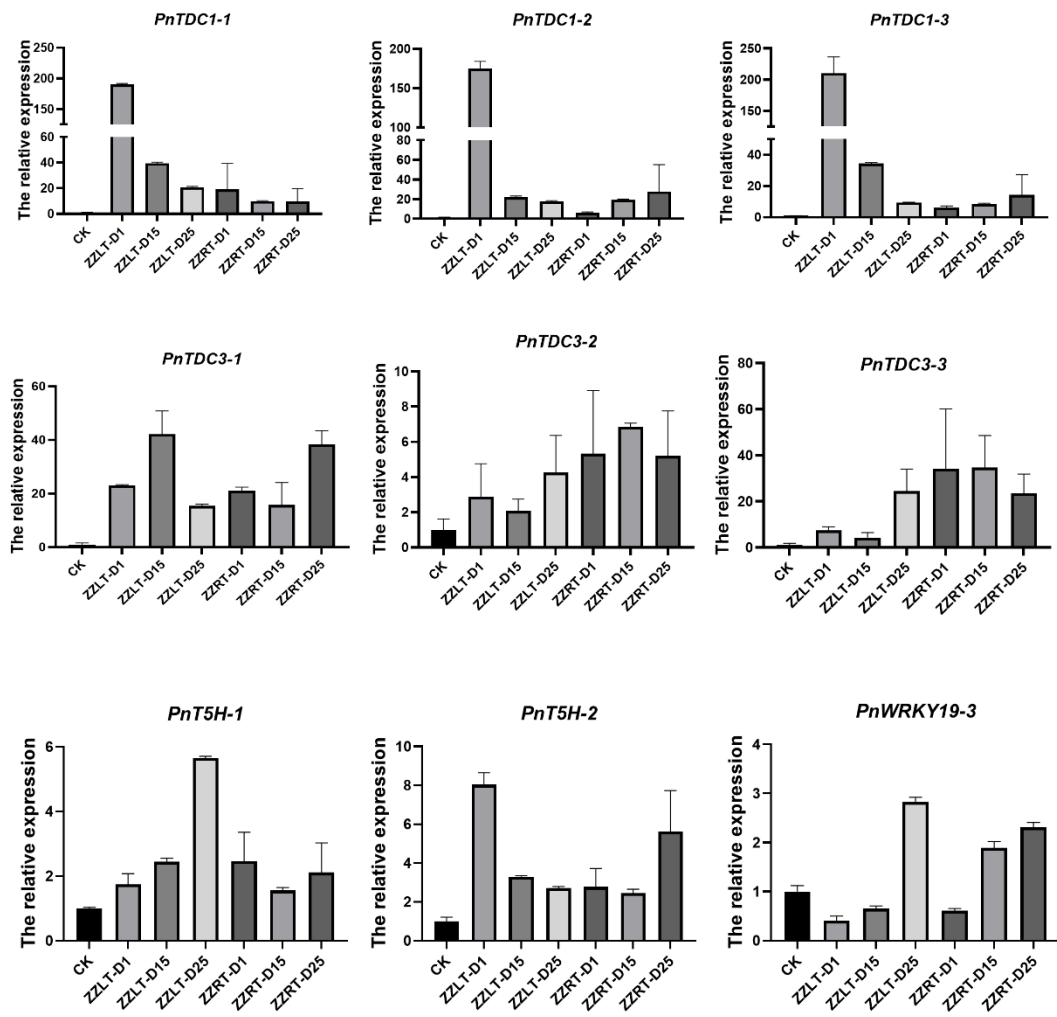

Figure S4 Gene expression analysis of *TDCs*, *T5Hs* and *PnWRKY19* under LT and RT treatments.

Supplement: Supplementary file 1 — Supplementary data to this article can be found online. [file forres-0025-0020-Supplementary.zip › 10.48130_forres-0025-0020-Suppl-FigureS4.pdf]

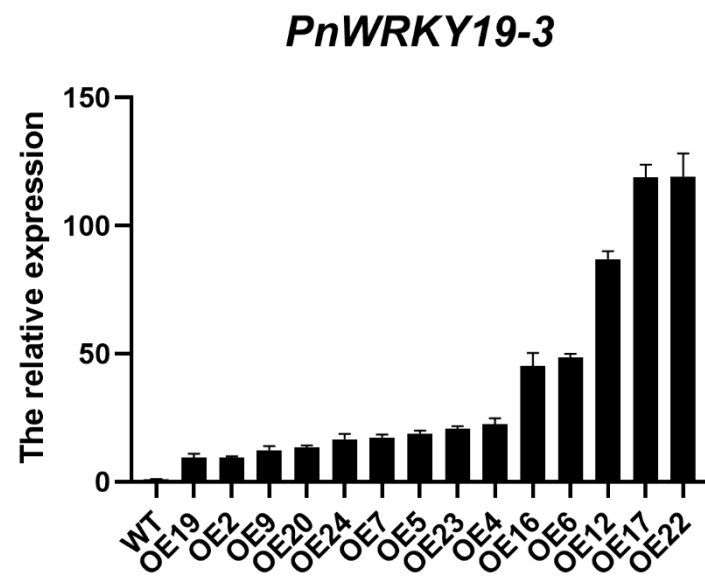

Figure S5 Gene expression analysis of different *PnWRKY19-3* OE rice lines.

Supplement: Supplementary file 1 — Supplementary data to this article can be found online. [file forres-0025-0020-Supplementary.zip › 10.48130_forres-0025-0020-Suppl-FigureS5.pdf]

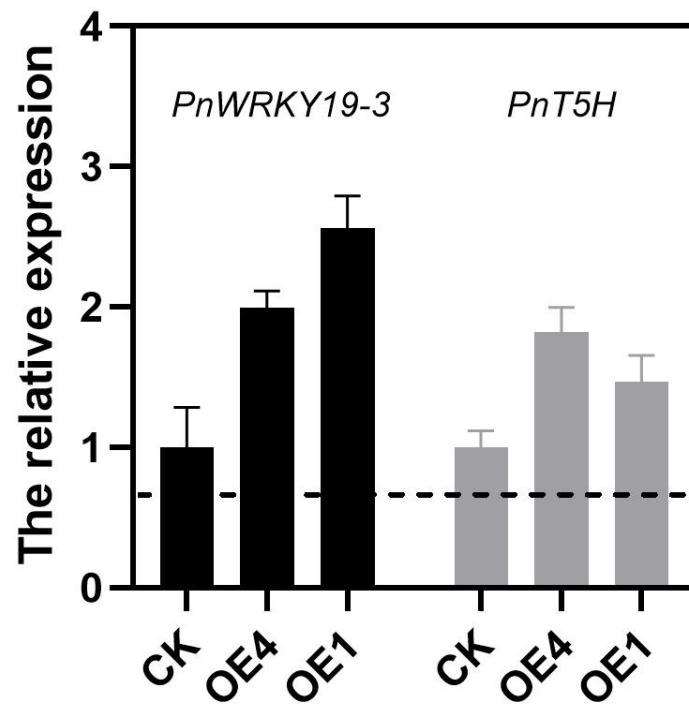

Figure S6. Gene expression analysis in *PnWRKY19-3* OE protoplasts in *Pnigra*.

Supplement: Supplementary file 1 — Supplementary data to this article can be found online. [file forres-0025-0020-Supplementary.zip › 10.48130_forres-0025-0020-Suppl-FigureS6.pdf]
